# Understanding sex differences in the allergic immune response to food

**DOI:** 10.3934/allergy.2022009

**Published:** 2022-07-06

**Authors:** McKenna S. Vininski, Sunanda Rajput, Nicholas J. Hobbs, Joseph J. Dolence

**Affiliations:** Department of Biology, University of Nebraska at Kearney, Kearney, NE, 68849

**Keywords:** peanut allergy, food allergy, tolerance, skin sensitization, lung sensitization, sex hormones, sex bias

## Abstract

Food allergies are of great public health concern due to their rising prevalence. Our understanding of how the immune system reacts to food remains incomplete. Allergic responses vary between individuals with food allergies. This variability could be caused by genetic, environmental, hormonal, or metabolic factors that impact immune responses mounted against allergens found in foods. Peanut (PN) allergy is one of the most severe and persistent of food allergies, warranting examination into how sensitization occurs to drive IgE-mediated allergic reactions. In recent years, much has been learned about the mechanisms behind the initiation of IgE-mediated food allergies, but additional questions remain. One unresolved issue is whether sex hormones impact the development of food allergies. Sex differences are known to exist in other allergic diseases, so this poses the question about whether the same phenomenon is occurring in food allergies. Studies show that females exhibit a higher prevalence of atopic conditions, such as allergic asthma and eczema, relative to males. Discovering such sex differences in allergic diseases provide a basis for investigating the mechanisms of how hormones influence the development of IgE-mediated reactions to foods. Analysis of existing food allergy demographics found that they occur more frequently in male children and adult females, which is comparable to allergic asthma. This paper reviews existing allergic mechanisms, sensitization routes, as well as how sex hormones may play a role in how the immune system reacts to common food allergens such as PN.

## 1. Introduction

Food allergies are very common, impacting approximately 10% of adults and 8% of children in the United States [1,2]. Amongst food allergies, peanut (PN) allergies represent one of the most common, severe, and persistent food allergies [3]. PN allergy remains a major medical problem as the prevalence of PN allergy is increasing rapidly [4]. Due to the tremendous burden that food allergies like PN allergy place on society, scientists have increased their studies in the last 10–15 years to better understand the immunological mechanisms involved in the development of food allergies. While more is understood about the disease, knowledge of how food allergies develop remains incomplete. Although many factors can influence allergic responses to food, including genetics, environment, microbiota, and diet, an outstanding question is what role sex hormones play in regulating these allergic responses [5,6]. Many diseases including cancer, autoimmunity, and allergic asthma have sex differences where males and females have different immune responses due to sex hormones [7-9]. Food allergies carry a similar trend. Data from epidemiological studies exhibit a sexual dimorphism in food allergy with women more likely at risk [9]. In support, a recent study showed that female children outnumber male children two to one in the incidence of PN allergy [10]. Furthermore, females outnumber male adults in the prevalence of PN allergy in both the United States and Mexico [10,11]. Due to the severe allergic reactions PN elicits and clinical relevance, this review will use PN as the example food allergen when discussing the immunological mechanisms driving allergic responses, sensitization routes, and the role sex hormones are playing in the development of allergic disease.

It is currently thought that exposure to PN via the airways or the skin promote sensitization to PN, while oral consumption, especially during infancy generates a tolerant environment to the allergen [12-14]. Since allergic asthma and inhalation-driven PN allergy share the same route of exposure to sensitize the immune system, this review will examine what can be learned from studying sex differences in allergic asthma and applied to our still nascent understanding of how sex hormones impact the development of food allergies like PN allergy.

Overall, this review discusses the allergic immune response to foods, using PN as the clinically relevant example food allergen. Routes of sensitization that lead to food allergy as well as how sex hormones may be influencing the allergic immune response via different immune cell subsets are explained. To conclude, we suggest that sex hormones are influencing the allergic immune response to food and make the case that this requires further investigation.

## 2. Types of hypersensitivities

The immune system offers protection from potential pathogens and monitors the body to maintain an individual’s health. However, the immune system can overreact and become harmful instead of beneficial. These types of immune responses are called hypersensitivities. There are four classified types of hypersensitivities. Type I hypersensitivities, more commonly known as allergies, are reactions to harmless environmental antigens found in substances such as foods, pollens, or drugs [15]. These types of reactions are driven by antigen-specific IgE antibodies [16]. For this reason, Type I hypersensitivities are commonly referred to as IgE-mediated allergies. Type II hypersensitivities are mediated by IgG or IgM that target cell surface antigens such as blood type antigens A or B [17]. Common Type II reactions are against red blood cells following an incompatible blood transfusion. In Type III hypersensitivity reactions, excess IgG or IgM bind foreign or self-antigen to generate immune complexes that deposit and activate complement system-driven inflammation. This inflammation leads to tissue damage. Common Type III reactions include drug-induced serum sickness, farmer’s lung, and systemic lupus erythematosus [18]. Type IV hypersensitivity reactions utilize T cells for delayed reactions, such as the reaction that occurs following exposure to poison ivy and metal jewelry [19]. This review paper expands on the importance of understanding the allergic mechanism of Type I hypersensitivity reactions (allergies) to food, using peanut (PN) allergy as the example food allergy. Such information is critical to design future therapeutic strategies to help those afflicted with food allergies.

## 3. Allergic immune response to foods using peanut as clinically relevant food allergen

Food allergies remain very prevalent in society, therefore it is crucial to understand the immunological response during an allergic reaction. The allergic reaction induced by the antigen, also referred to as an allergen, can range from mild urticaria (skin rash) to severe anaphylactic reactions that are sometimes fatal. Allergic individuals become sensitized when their immune system reacts to a certain food allergen, and this sensitization can be based on various factors such as genetics, environment, and microbiota [20]. Other factors, such as hormones and dietary choices, are likely to play a role in either offering protection or promoting sensitization to foods [5,21]. Treating and studying allergies can be complicated by the variability that exists amongst individuals [22].

To better understand how the various factors impacting the development of food allergies impact sensitization to a particular food allergen, it is critical to understand the routes of sensitization that lead to food allergy as well as how sex hormones may be influencing the allergic immune response via different immune cell subsets. Due to its prevalence, persistence, severity, as well as our understanding of the routes of sensitization from clinical trials and mouse models, this review paper will use PN allergy as the clinically relevant example of food allergy.

PN allergy is the most prevalent food allergy that impacts approximately 2% of children and 2% of adults in the United States [3,23]. The incidence of PN allergy among children in the United States increased 5-fold from 0.4% in 1997 to 2.0% in the most recent national survey [4]. Approximately 59% of PN allergic individuals have had a severe allergic reaction and 50% have visited the emergency room in their lifetime due to the allergic reaction [24]. Most PN-allergic children experience their first allergic reaction upon first oral ingestion [25]. The Learning Early About Peanut Allergy (LEAP) study showed early introduction of PN into the diet prevented the development of clinical PN allergy among children at high risk, suggesting early oral exposure may induce oral tolerance [26]. PN is known to be readily detectable in household dust and a greater percentage of children in LEAP’s PN avoidance group developed elevated titers of PN-specific IgE antibody, suggesting sensitization to environmental PN allergens (e.g., 3~100 μg PN/gram dust) [27,28]. Children in the United Kingdom (UK) have a much higher prevalence of PN allergy whereas there were very few cases in Israel [14]. Data show that Israeli children consume PN products at a significantly higher level during infancy than their UK counterparts, which is hypothesized to severely decrease the incidence of PN allergy in Israel [14]. These relevant data regarding PN allergy indicate that early oral exposure leads to tolerance, the default state of the immune system not to react to the food we ingest.

**Figure 1.**
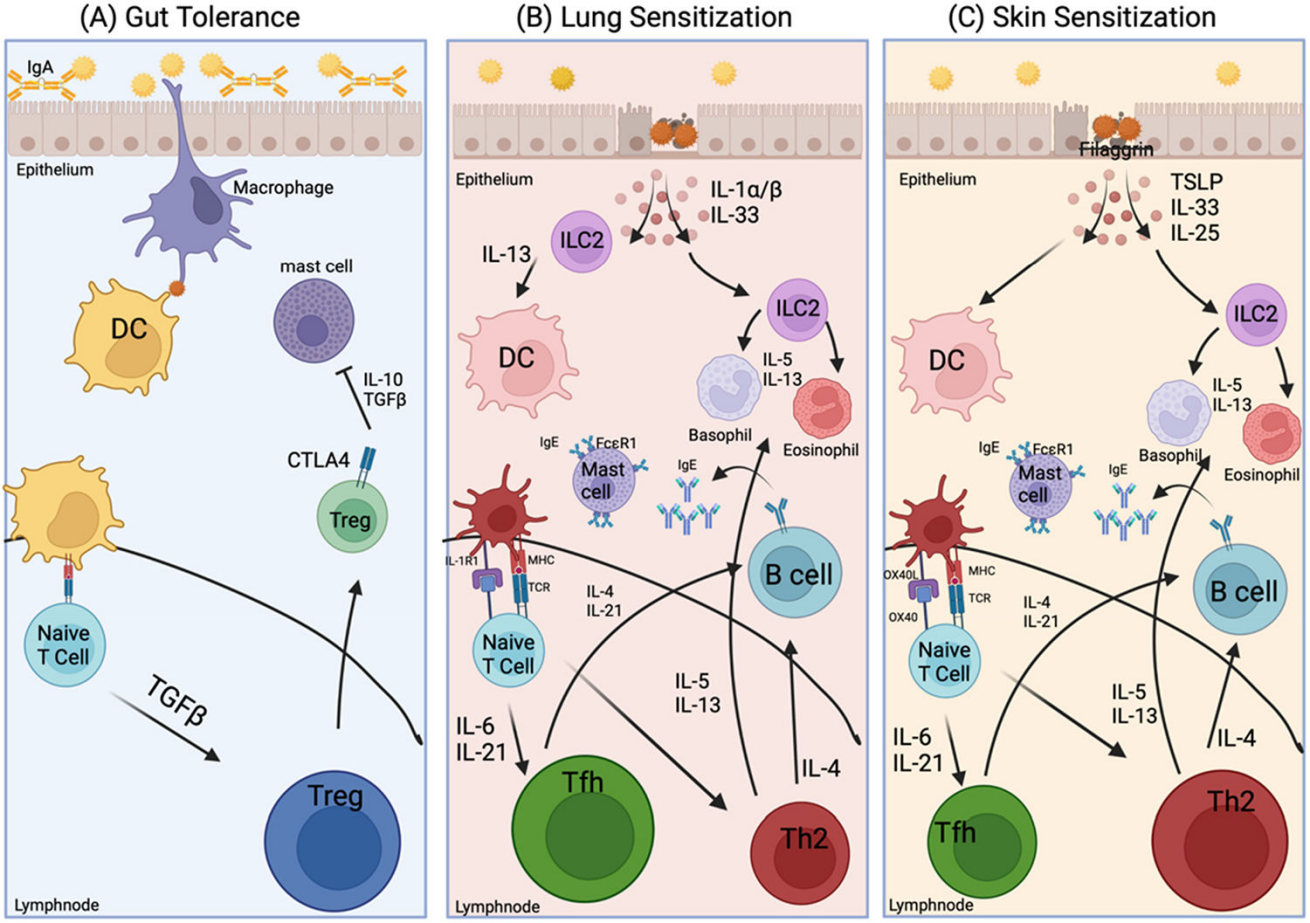
Environmental sensitization to food allergens, using peanut (PN) as the example food allergen, through the lungs and skin. (A) The immune system promotes tolerance by patrolling the intact gut barrier with diverse microbiota and active Immunoglobulin A (IgA). (B,C) Sensitization can occur through the lungs and skin. (B) Lungs: Type 2 innate lymphoid cells (ILC2s) are stimulated by cytokines IL-1α (and possibly IL-33) released from allergen-exposed epithelial cells. These ILC2s secrete IL-13 to activate dendritic cells (DCs) to trigger a T follicular helper (Tfh) cell-mediated, IgE antibody response against PN. IL-4 secreted by Tfh and to a lesser extent T helper 2 (Th2) cells promote B cell receptor class switching to Immunoglobulin E (IgE). IgE is then secreted by plasma B cells and bind to FcεR1 on mast cells. Once mast cells have bound the allergen-specific IgE, the individual is considered sensitized to the allergen. (C) Skin: The skin can lose integrity via a loss of function mutation in the filaggrin gene. Sensitization is promoted by disrupted epithelial barrier and the perpetuation of Th2-promoting cytokines. DCs are activated by epithelial cytokines TSLP, IL-33 and IL-25 that promote expression of OX40L. OX40/OX40L signaling stimulates naïve T cells to mature into Th2 cells. Th2 cells secrete IL-4 to promote the production of PN-specific IgE, which as described above, binds mast cells to cause sensitization to the food allergen. This figure was created using BioRender.com.

Tolerance in the gut is established by a functional gut epithelial barrier. Resident macrophages monitor the gut epithelial barrier for pathogenic invasion. If a harmless peptide from food passes through the barrier, a dendritic cell displays it to a naïve CD4+ T cell which promotes maturation of regulatory T cells (Tregs) via transforming growth factor (TGF)-β and retinoic acid (RA) (Figure 1A) [29]. These Tregs then patrol the environment downregulating mast cells. Mast cells are highly granular cells that reside in mucosal tissues. IgE antibodies that are generated by B cells upon sensitization to a food allergen bind to the surface of mast cells. A tolerant response does not allow crosslinking of IgE, thus preventing the activation of mast cells. In this scenario, mast cells do not release granules and the inflammatory environment that causes the clinical symptoms of food allergy is avoided [30]. As previously stated, introducing foods known to cause allergic reactions during infancy has been shown to promote tolerance in clinical studies [14,31]. This tolerogenic environment regulates the response to harmless peptides (Figure 1A), while maintaining protection against harmful pathogenic agents.

During the state of non-tolerance, called sensitization, the immune system recognizes harmless antigen(s) in food as harmful. In an allergic reaction, the goal of the immune system is to rid the body of the antigen, which causes a reaction that manifests itself as clinical symptoms. An immune environment susceptible to promoting allergic sensitization is marked by a dysregulated epithelial barrier [32]. In this scenario, macrophages fail to patrol the barrier to regulate the response. Harmless peptides enter incorrectly and are recognized as harmful (Figure 1B,C). This entrance stimulates the release of cytokines by barrier epithelial cells associated with allergic responses: interleukin (IL)-25, IL-33, thymic stromal lymphopoietin (TSLP), and even IL-1α and IL-1β [12,33]. Studies from the past decade have provided insight into what occurs following the release of the cytokines from the damaged epithelium that was exposed to foods [6,34]. The release of these cytokines initiates a feedback loop leading to sensitization and production of the food allergen-specific IgE that bind to the surface of mast cells via the FcεR1, activating these cells to react upon subsequent exposure to that particular food [35]. In contrast to tolerance mediated via the gut, non-oral routes of PN exposure have been shown to induce allergic response to PN. Sensitization to PN can be induced through the airways and skin in mice [12,36,37]. Of note, sensitization through the skin and airways can be abrogated by feeding the mice PN [38]. This data suggests that non-oral exposure to PN via the airways, skin, or both, in the absence of early PN ingestion causes initial sensitization to the allergen (Figure 1B,C).

Inhalation is a likely route of sensitization because PN is commonly found in household dust and is biologically active [27,28,39,40]. This route of sensitization can stimulate the immune response with every breath instead of relying on contact through the skin. We showed that PN exposure through the airways elicited PN sensitization in mice, and upon PN challenge, anaphylaxis occurs [12]. This study also showed that signaling through IL-1R1 was critical to mount PN-specific T follicular helper (Tfh) cell response. In the airways, we found that Tfh cells and to a lesser extent, type 2 T cell (Th2), secrete IL-4 to promote B cell class switching to become allergen-specific IgE producing B cells [12]. Additional studies by Krempski et al. showed that following airway exposure to PN, the epithelium-derived cytokines IL-1α (and possibly IL-33) stimulates type 2 innate lymphoid cells (ILC2s) to secrete IL-13 to activate DCs that work to trigger a Tfh-mediated, PN-specific IgE antibody response (Figure 1B) and that this response can be inhibited by stimulating CTLA-4-expressing Tregs following ingestion of PN [36,38]. In addition, they showed that IL-1α alone can induce IL-13 production from ILC2s and signaling through IL-1R1 (the receptor for IL-1α and IL-1β) is critical for IL-13 release. Collectively, this data strongly suggests that IL-1α (and maybe even IL-1β which also signals via IL-1R1) and perhaps IL-33 via the activation of IL-1R1+ ILC2s and ST2+ ILC2s, respectively, work to initiate sensitization to PN via the airways. While more studies need to be done to understand how food allergens stimulate respiratory tract-mediated immune responses, it is becoming clear from mouse models and human cell-based studies that the airways are a plausible route of sensitization [39].

Sensitization to PN can also occur via the skin. Mice exposed to PN antigens on healthy skin generated a PN-specific antibody response that enabled an anaphylactic response to be induced upon rechallenge, providing strong evidence that PN allergy can develop via skin exposure [37]. Further evidence of skin sensitization can be found when individuals who have epithelial dysfunction are examined [41-44]. Eczema or atopic dermatitis is a common comorbidity of PN allergy due to a loss-of-function mutation in the *filaggrin* gene which results in a loss of skin barrier integrity [41-44]. Disruption in the integrity of the barrier leads to release of TSLP, IL-33, and IL-25 in the epidermal layer to promote allergic sensitization via dysregulation of Treg cells due to the microenvironmental disruption [45,46]. These cytokines work to activate innate immune cells such as ILC2s, DCs, and basophils. DCs stimulate naïve T cells to become Th2 cells via OX40-OX40L signals [47]. ILC2s and basophils further amplify the Th2 response by secreting Th2 cytokines [46]. The collective effect of this cellular response is to promote a robust humoral type 2 response in the skin. IL-4 in the environment produced by Th2 cells and innate cells stimulate B cells to class switch to generate PN-specific IgE (Figure 1C). As previously discussed, this IgE binds mast cells and leads to sensitization to PN. At this point, subsequent exposure to PN causes activation of mast cells and the clinical symptoms associated with PN allergy. Similar to inhalation, exposure through the skin is a likely route of sensitization due to the biological activity of PN in the environment [27,28,39,40]. Taken together, the scientific evidence strongly suggests that sensitization to food allergens can occur via skin exposure. In sharp contrast, while sensitization can occur in both lung and skin epithelium, it is current thought that exposure to PN through the gut promotes oral tolerance, preventing allergic reactions in healthy individuals [48,49].

Our knowledge on the influence of sex hormones on immune function in allergic disease has been mainly examined in allergic asthma [50,51]. To date, a significant gap in the literature exists in understanding the role of sex hormones in driving allergic responses to food. Due to the strong influence of sex hormones on immune cells that participate in other allergic and non-allergic disease pathways, it makes logical sense to investigate the influence of sex hormones on the development of food allergies, which we will now discuss.

## 4. The influence of sex hormones on immune function and allergic disease gives insight into impact on food allergy

Sex differences exist in the ability of males and females to mount immune responses to common pathogens, such as bacteria and viruses. Females are better protected than males because they have a more reactive immune system [52,53]. Not only have females been shown to clear pathogenic infections quicker, they also can mount a more robust immune response to vaccinations than their male counterparts (Table 1) [54-56]. However, this enhanced reactivity can be detrimental during allergy and autoimmune reactions [57].

Several pieces of evidence show that males and females develop allergies differently during childhood and these sex differences often change during adolescence. Before puberty, males display greater amounts of general atopic symptoms such as skin reactions against one or more allergens than females [58]. Additionally, there is known to be a male predominance to asthma before puberty, which is reversed to female predominance post-puberty [58-60]. Furthermore, adolescent females have shown to experience more respiratory allergies and asthma [58,61]. Moreover, women have reported premenstrual worsening of asthma symptoms and changes in asthma control during pregnancy [62-67]. These data suggest that hormonal changes brought about by puberty and pregnancy carry a strong influence on allergy and asthma. While this sex difference has been best studied in asthma, evidence is accumulating that food allergies also display similar sex differences [68].

Studies dating back to the early 1980s have described a sex bias in food allergy [69,70]. Those studies, along with others conducted in the 1990s and 2000s, demonstrated a sex ratio of 60:40 in adult women to men suffering from food allergy [71-76]. More recently, a literature review published in 2009 described the prevalence of IgE-mediated food allergy in those under 18 years old as favoring males over females at a 1.8 to 1 ratio [77]. Data, summarized in a recent review by Pali-Schöll and Jensen-Jarolim, clearly show that during childhood, boys are impacted more than girls regarding the development of food allergy [68]. Of note, similar to asthma, a shift in prevalence towards females occurs by the time an individual reaches adulthood. From around the age of 18 until menopause, women are more likely to have a food allergy (woman to man ratio of 1:0.53) [68]. Interestingly, sex differences between the frequency of males and females who develop PN allergy in childhood appear relatively stable into adulthood. A recent study of US adults allergic to PN showed that female adults were twice as likely to develop PN allergy during their childhoods than their male counterparts [10]. The two-fold difference favoring females with PN allergy was maintained into adulthood in the United States and Mexico [10,11]. Whether PN allergy is unique in its ability among food allergies to avoid being impacted by the onset of puberty requires more examination. Collectively, this data strongly suggests that allergic immune reactions to food allergens are sensitive to sex hormones and that studying such sex differences and the mechanism impacted by sex hormones are very clinically relevant.

Gaps exist in our current knowledge about how sex hormones mechanistically impact allergic responses to food. Examining what is known about how sex hormones impact immune cells under both non-disease and disease states such as asthma and autoimmunity gives insight into how the male and female immune system will respond differently to food allergens.

Both cell-mediated and humoral responses are known to be enhanced by estrogens, which provides an explanation to why females react stronger to immune stimuli (Table 1) [52,78]. Additionally, levels of estrogens fluctuate throughout the menstrual cycle, which may influence the immune response during menses, as well as pre- and post-menopause, and during pregnancy [62,79,80]. Estrogens use their receptors, ERα and ERβ, to signal intracellularly to carry out their function [81,82]. ERs are commonly expressed in a variety of immune cells, including lymphocytes, macrophages, eosinophils, basophils, DCs, mast cells, as well as B and T cells [54,80,83]. Both ERs can work to influence immune responses at the cellular level [81,82,84]. Although the effect of estrogen on T cells is complex, data suggest that Th1 responses are promoted under low estrogen conditions and Th2 responses are promoted under high estrogen conditions [84]. In support, estrogens may enhance the function of antigen presenting cells (APCs) to develop allergic disease (possibly through promoting Th2 responses), promote B cell class switching to IgE and the degranulation of mast cells (reviewed in [85]). Estrogen has been shown in a mouse model to promote airway inflammation through the Th2-promoting cytokine IL-33 (Table 1) [86]. These effects provide a plausible explanation as to why females suffer from more asthma and allergic disease that are driven by classical Type 2 (Th2-based) responses.

Looking beyond allergy is helpful to obtain additional insight into the effect of estrogen on conditions of immune over reactivity. About 90% of lupus patients are women and this marked sex bias is believed to be caused, in part, by estrogen. Inhibiting ER function, specifically ERα, in mouse models of lupus was beneficial for disease pathogenesis [87]. Estrogens increase B and T cell activation and autoantibody production (Table 1) [84,87]. Overall, ERs make females more susceptible to over reactivity from their immune systems compared to males. Due to this, females are at greater risk for developing conditions such as asthma, allergies, and autoimmune diseases such as lupus.

Androgen receptor (AR) signaling influences the immune response differently than ERs. AR signaling suppresses immune responses, which provides a reason as to why males are known to be more susceptible to common pathogens (Table 1) [91-93]. Androgens carry out their function via a nuclear AR. ARs begin to be expressed in hematopoietic progenitors, and as such, they are expressed by immune cells found in the bone marrow, thymus, and spleen [93,94]. Due to their wide expression on immune cells, AR signaling exerts a broad, immunomodulatory effect on immune cells, and for the most part, male immune cells are less reactive than their female counterparts (reviewed in [9,52,91,93]). Of note, testosterone has been shown to suppress conventional DC (cDCs) responses. Male plasmacytoid DCs (pDCs), a potent producer of type I interferons against viral infections, produced significantly less type I interferons against HIV-1 infection, elicited weaker HIV-specific CD8+ T cell responses, which resulted in a higher HIV titer in male compared to female patients [95]. Interestingly, the removal of androgens revealed an increase of MHC Class II and co-stimulatory molecule expression on conventional DCs in lymph nodes [93]. In adaptive immunity, androgens seem to exert an overall inhibitory effect on Th1 and Th2 responses (reviewed in [93]). Tregs are also increased in males [96], promoting a more regulatory and less reactive environment. B cell numbers are also influenced by testosterone levels. More B cells are recorded in men with low testosterone, while high testosterone is correlated with a poor antibody response to vaccination [9]. In general, due to the impact of androgens, adult males have lower B cell responses and lower antibody responses to foreign antigens than adult females (Table 1) [57]. While the male immune environment stifles immune responses leading to more susceptibility to common pathogens, it allows adult males to be less susceptible to allergy, asthma, and autoimmune disease.

Testosterone has been shown to reduce airway inflammation induced by house dust mite and *Alternaira alternate* fungal extract in murine models via the negative regulation of ILC2s [8,89,90,97]. The androgen dehydroepiandrosterone (DHEA) has worked to increase lung function thereby decreasing asthma symptoms in clinical studies [98-100]. Furthermore, a recent study documented that AR signaling increased Treg suppression and decreased IL-33 production from airway epithelial cells using mouse models of airway inflammation (Table 1). The same study also showed that dihydrotestosterone decreased fungal extract-induced IL-33 secretion from human bronchial epithelial cells [88]. The impact of androgen signaling on modulating immune responses during asthma development helps give insight into why males experience less asthmatic burden when compared to females.

Androgens also offer protection against developing autoimmunity [8,9,80,101-104]. Androgens are known to play a role in offering protection against males developing several autoimmune disorders including lupus, multiple sclerosis (MS), and rheumatoid arthritis [9,91]. Taken together, the impact of androgens on autoimmunity, asthma, in fighting pathogens, and responding to vaccination makes it clear that male sex hormones play an important role in inhibiting immune reactions.

While there is increasingly more research being done on sex differences in disease, there is still much to be learned, specifically to understand how mechanistically male and female immune systems respond differently during the development of food allergy. Hormones most likely play a role in driving mechanistic differences on the cellular and molecular levels, either directly or indirectly, given that sex biases in food allergy have been documented. This hormonal influence likely affects allergic responses to food allergens like PN. Therefore, it is imperative to examine how sex hormones influence the development of these allergic immune responses to food using mouse and human studies in asthma and autoimmunity as a guide. Such studies will allow for the acquisition of knowledge that will allow us to develop a greater understanding of allergic immune mechanisms against PN and other foods. This will allow for potential sex-specific therapeutic strategies to be designed and allow for better management of food allergy patients.

## 5. Conclusions

Food allergies cause a severe burden on the allergic individual, their family, and community. Allergies continue to be on the rise despite increasing awareness and prevalence. In this review, we used PN as the example of a clinically relevant food allergen to explain what is known about the immunological mechanism driving the development of allergic reactions to food. Currently, there are two recognized ways an individual can become sensitized to PN allergens, through the skin and lungs. These sensitization routes stem from environmental exposures, likely to PN found in household dust. Exposure to PN allergens via dysregulated barriers in both the skin and lungs in the absence of established oral tolerance likely drives sensitization to PN and the development of allergic disease. Although progress has been made in the past decade to understand the allergic mechanisms of IgE-mediated food allergy, it is still unknown why certain individuals develop food allergies. Sex differences in food allergy have been documented in epidemiological studies. Future studies need to investigate the immunological mechanisms behind these differences. Understanding how sex hormones are influencing the allergic immune response to PN and other foods could give insight into how to develop novel treatments to reduce the severity and improve the quality of life for allergic individuals. Not only is understanding the allergic mechanism important for treatments, but also to design new prevention strategies. Such knowledge may allow for hormone levels or signaling pathways to be regulated with the overall goal being prevention of the allergic reaction to foods from occurring and ultimately, to slow the growing epidemic of food allergy.

## Figures and Tables

**Table 1.** Sex difference on immune function and allergic disease.

	Male	Female	Reference
Autoimmunity	Androgens ↓ immune reactivity	Estrogens ↑ immune reactivity	[84,87,88]
		Estrogens ↑ autoantibody	
Infection	Less reactive to common pathogens	More reactive to common pathogens	[52,89]
	↑ susceptibility	↓ susceptibility	
	↓ MHCII & costimulatory molecules on cDCs	↑ pro-inflammatory	
	↓ production of Type I IFNs from pDCs	↑ cell-mediated response	
	↓ cell-mediated response		
	↑ cell-mediated response		
Vaccination	↑ Androgens lead to:	↑ Estrogens lead to:	[57,89]
	↓ B cell activity	↑ humoral response	
	↓ memory B cells	↑ memory B cells	
Allergy	↓ Allergic asthma	↑ Allergic asthma	[58-61]
	↑ Androgens lead to:	↑ Estrogens lead to:	[84-86,88-90]
	↑ Treg; ↓ IL-33; ↓ ILC2s	↑ Th2; ↑ IL-33; ↑ ILC2s	
	Food allergy bias pre-puberty	Food allergy bias post-puberty	[77]

## References

[1] Jiang J, Bushara O, Ponczek J, (2018) Updated pediatric peanut allergy prevalence in the United States. Ann Allerg Asthma Im 121: S14. 10.1016/j.anai.2018.09.042

[2] Gupta RS, Warren CM, Smith BM, (2019) Prevalence and severity of food allergies among US adults. JAMA Netw Open 2: e185630–e185630. 10.1001/jamanetworkopen.2018.5630 30646188 PMC6324316

[3] Lieberman JA, Gupta RS, Knibb RC, (2021) The global burden of illness of peanut allergy: A comprehensive literature review. Allergy 76: 1367–1384. 10.1111/all.14666 33216994 PMC8247890

[4] Togias A, Cooper SF, Acebal ML, (2017) Addendum guidelines for the prevention of peanut allergy in the United States: Report of the National Institute of Allergy and Infectious Diseases—sponsored expert panel. J Allergy Clin Immun 139: 29–44. 10.1016/j.jaci.2016.10.010 28065278 PMC5226648

[5] Dolence JJ, Kita H (2020) Allergic sensitization to peanuts is enhanced in mice fed a high-fat diet. AIMS Allerg Immu 4: 88–99. 10.3934/Allergy.2020008 PMC10831907 38304556

[6] Zhu TH, Zhu TR, Tran KA, (2018) Epithelial barrier dysfunctions in atopic dermatitis: a skin–gut–lung model linking microbiome alteration and immune dysregulation. Brit J Dermatol 179: 570–581. 10.1111/bjd.16734 29761483

[7] Zhu Y, Shao X, Wang X, (2019) Sex disparities in cancer. Cancer Lett 466: 35–38. 10.1016/j.canlet.2019.08.017 31541696

[8] Fuseini H, Newcomb DC (2017) Mechanisms driving gender differences in asthma. Physiol Behav 176: 139–148. 10.1007/s11882-017-0686-1 28332107 PMC5629917

[9] Gubbels Bupp MR, Jorgensen TN (2018) Androgen-induced immunosuppression. Front Immunol 9: 794. 10.3389/fimmu.2018.00794 29755457 PMC5932344

[10] Warren C, Lei D, Sicherer S, (2021) Prevalence and characteristics of peanut allergy in US adults. J Allergy Clin Immun 147: 2263–2270. 10.1016/j.jaci.2020.11.046 33579526 PMC12341317

[11] Hernández-Colín DD, Fregoso-Zúñiga E, Morales-Romero J, (2019) Peanut allergy among Mexican adults with allergic respiratory diseases: prevalence and clinical manifestations. Rev Alerg Mex 66: 314–321. 10.29262/ram.v66i3.619 31606015

[12] Dolence JJ, Kobayashi T, Iijima K, (2018) Airway exposure initiates peanut allergy by involving the IL-1 pathway and T follicular helper cells in mice. J Allergy Clin Immun 142: 1144–1158. 10.1016/j.jaci.2017.11.020 29247716 PMC6002896

[13] Du Toit G, Roberts G, Sayre PH, (2015) Randomized trial of peanut consumption in infants at risk for peanut allergy. N Engl J Med 372: 803–813. 10.1056/NEJMoa1414850 25705822 PMC4416404

[14] Du Toit G, Katz Y, Sasieni P, (2008) Early consumption of peanuts in infancy is associated with a low prevalence of peanut allergy. J Allergy Clin Immun 122: 984–991. 10.1016/j.jaci.2008.08.039 19000582

[15] Platts-Mills TAE (2001) The role of immunoglobulin E in allergy and asthma. Am J Resp Crit Care 164: S1–S5. 10.1164/ajrccm.164.supplement_1.2103024 11704610

[16] Fuller RW, Morris PK, Richmond R, (1986) Immunoglobulin E-dependent stimulation of human alveolar macrophages: Significant in type 1 hypersensitivity. Clin Exp Immunol 65: 416–426. 2947765 PMC1542321

[17] Viel S, Pescarmona R, Belot A, (2018) A case of type 2 hypersensitivity to rasburicase diagnosed with a Natural Killer cell activation assay. Front Immunol 9: 110. 10.3389/fimmu.2018.00110 29434608 PMC5796899

[18] Eggleton P (2013) Hypersensitivity: Immune Complex Mediated (Type III), 1 Ed., eLS.

[19] Uzzaman A, Cho SH (2012) Classification of hypersensitivity reactions. Allergy Asthma Proc 33: 96–99. 10.2500/aap.2012.33.3561 22794701

[20] Sampson HA, O’Mahony L, Burks AW, (2018) Mechanisms of food allergy. J Allergy Clin Immun 141: 11–19. 10.1016/j.jaci.2017.11.005 29307410

[21] Pali-Schöll I, Herzog R, Wallmann J, (2010) Antacids and dietary supplements with an influence on the gastric pH increase the risk for food sensitization. Clin Exp Allergy 40: 1091–1098. 10.1111/j.1365-2222.2010.03468.x 20214670 PMC2999750

[22] Van Erp FC, Knulst AC, Meijer Y, (2014) Standardized food challenges are subject to variability in interpretation of clinical symptoms. Clin Transl Allergy 4: 1–6. 10.1186/s13601-014-0043-6 25493173 PMC4260179

[23] Dyer AA, Rivkina V, Perumal D, (2015) Epidemiology of childhood peanut allergy. Allergy Asthma Proc 36: 58–64. 10.2500/aap.2015.36.3819 25562557

[24] Gupta RS, Warren C, Smith BM, (2019) The public health impact of parent-reported childhood food allergies in the United States. Pediatrics 144: S28. 10.1542/peds.2019-2461PP PMC6317772 30455345

[25] Sicherer SH, Morrow EH, Sampson HA (2000) Dose-response in double-blind, placebo-controlled oral food challenges in children with atopic dermatitis. J Allergy Clin Immun 105: 582–586. 10.1067/mai.2000.104941 10719311

[26] Du Toit G, Roberts G, Sayre PH, (2015) Randomized trial of peanut consumption in infants at risk for peanut allergy. N Engl J Med 372: 803–813. 10.1056/NEJMoa1414850 25705822 PMC4416404

[27] Smeekens JM, Immormino RM, Balogh PA, (2019) Indoor dust acts as an adjuvant to promote sensitization to peanut through the airway. Clin Exp Allergy 49: 1500–1511. 10.1111/cea.13486 31444814 PMC7171466

[28] Trendelenburg V, Ahrens B, Wehrmann AK, (2013) Peanut allergen in house dust of eating area and bed—A risk factor for peanut sensitization? Allergy 68: 1460–1462. 10.1111/all.12226 24351066

[29] Anvari S, Miller J, Yeh CY, (2019) IgE-mediated food allergy. Clin Rev Allerg Immu 57: 244–260. 10.1007/s12016-018-8710-3 30370459

[30] Gri G, Piconese S, Frossi B, (2008) CD4+CD25+ regulatory T cells suppress mast cell degranulation and allergic responses through OX40-OX40L interaction. Immunity 29: 771–781. 10.1016/j.immuni.2008.08.018 18993084 PMC2590499

[31] Tordesillas L, Berin MC (2018) Mechanisms of oral tolerance. Clin Rev Allerg Immu 55: 107–117. 10.1007/s12016-018-8680-5 PMC6110983 29488131

[32] Goleva E, Berdyshev E, Leung DYM (2019) Epithelial barrier repair and prevention of allergy. J Clin Invest 129: 1463–1474. 10.1172/JCI124608 30776025 PMC6436854

[33] Eiwegger T, Hung L, San Diego KE, (2019) Recent developments and highlights in food allergy. Allergy 74: 2355–2367. 10.1111/all.14082 31593325

[34] Platts-Mills TAE, Woodfolk JA (2011) Allergens and their role in the allergic immune response. Immunol Rev 242: 51–68. 10.1111/j.1600-065X.2011.01021.x 21682738

[35] Heib V, Becker M, Taube C, (2008) Advances in the understanding of mast cell function. Brit J Haematol 142: 683–694. 10.1111/j.1365-2141.2008.07244.x 18513284

[36] Krempski JW, Kobayashi T, Iijima K, (2020) Group 2 innate lymphoid cells promote development of T follicular helper cells and initiate allergic sensitization to peanuts. J Immunol 204: 3086–3096. 10.4049/jimmunol.2000029 32366582 PMC7309436

[37] Tordesillas L, Goswami R, Benedé S, (2014) Skin exposure promotes a Th2-dependent sensitization to peanut allergens. J Clin Invest 124: 4965–4975. 10.1172/JCI75660 25295541 PMC4347216

[38] Krempski JW, Lama JK, Iijima K, (2022) A mouse model of the “LEAP” study reveals a role for CTLA-4 in preventing peanut allergy induced by environmental peanut exposure. J Allergy Clin Immun In press. 10.1016/j.jaci.2022.02.024 PMC9378358 35288169

[39] Kulis MD, Smeekens JM, Immormino RM, (2021) The airway as a route of sensitization to peanut: An update to the dual allergen exposure hypothesis. J Allergy Clin Immun 148: 689–693. 10.1016/j.jaci.2021.05.035 34111450 PMC8429226

[40] Brough HA, Santos AF, Makinson K, (2013) Peanut protein in household dust is related to household peanut consumption and is biologically active. J Allergy Clin Immun 132: 630–638. 10.1016/j.jaci.2013.02.034 23608730

[41] Flohr C, England K, Radulovic S, (2010) Filaggrin loss-of-function mutations are associated with early-onset eczema, eczema severity and transepidermal water loss at 3 months of age. Brit J Dermatol 163: 1333–1336. 10.1111/j.1365-2133.2010.10068.x 21137118

[42] Brough HA, Simpson A, Makinson K, (2014) Peanut allergy: Effect of environmental peanut exposure in children with filaggrin loss-of-function mutations. J Allergy Clin Immun 134: 867–875. 10.1016/j.jaci.2014.08.011 25282568 PMC4188983

[43] Venkataraman D, Soto-Ramírez N, Kurukulaaratchy RJ, (2014) Filaggrin loss-of-function mutations are associated with food allergy in childhood and adolescence. J Allergy Clin Immun 134: 876–882. 10.1016/j.jaci.2014.07.033 25174864 PMC4186905

[44] Brown SJ, Asai Y, Cordell HJ, (2011) Loss-of-function variants in the filaggrin gene are a significant risk factor for peanut allergy. J Allergy Clin Immun 127: 661–667. 10.1016/j.jaci.2011.01.031 21377035 PMC3081065

[45] Wang YH (2016) Developing food allergy: A potential immunologic pathway linking skin barrier to gut. F1000Research 5: 1–8. 10.12688/f1000research.9497.1 PMC5105878 27853507

[46] Tordesillas L, Berin MC, Sampson HA (2017) Immunology of food allergy. Immunity 47: 32–50. 10.1016/j.immuni.2017.07.004 28723552

[47] Ito T, Wang YH, Duramad O, (2005) TSLP-activated dendritic cells induce an inflammatory T helper type 2 cell response through OX40 ligand. J Exp Med 202: 1213–1223. 10.1084/jem.20051135 16275760 PMC2213234

[48] Staden U, Rolinck-Werninghaus C, Brewe F, (2007) Specific oral tolerance induction in food allergy in children: Efficacy and clinical patterns of reaction. Allergy 62: 1261–1269. 10.1111/j.1398-9995.2007.01501.x 17919140

[49] Logan K, Du Toit G, Giovannini M, (2020) Pediatric allergic diseases, food allergy, and oral tolerance. Annu Rev Cell Dev Bi 36: 511–528. 10.1146/annurev-cellbio-100818-125346 32634325

[50] Mccallister JW, Mastronarde JG (2008) Sex differences in asthma. J Asthma 45: 853–861. 10.1080/02770900802444187 19085573

[51] Schatz M, Clark S, Camargo CA (2006) Sex differences in the presentation and course of asthma hospitalizations. Chest 129: 50–55. 10.1378/chest.129.1.50 16424412

[52] Klein SL, Flanagan KL (2016) Sex differences in immune responses. Nat Rev Immunol 16: 626–638. 10.1038/nri.2016.90 27546235

[53] Furman D, Hejblum BP, Simon N, (2014) Systems analysis of sex differences reveals an immunosuppressive role for testosterone in the response to influenza vaccination. P Natl Acad Sci USA 111: 869–874. 10.1073/pnas.1321060111 PMC3896147 24367114

[54] Keselman A, Heller N (2015) Estrogen signaling modulates allergic inflammation and contributes to sex differences in asthma. Front Immunol 6: 568. 10.3389/fimmu.2015.00568 26635789 PMC4644929

[55] Gilliver SC (2010) Sex steroids as inflammatory regulators. J Steroid Biochem 120: 105–115. 10.1016/j.jsbmb.2009.12.015 20045727

[56] Afify SM, Pali-Schöll I (2017) Adverse reactions to food: The female dominance—A secondary publication and update. World Allergy Organ J 10: 1–8. 10.1186/s40413-017-0174-z PMC5746020 29308110

[57] Fink AL, Klein SL (2018) The evolution of greater humoral immunity in females than males: implications for vaccine efficacy. Curr Opin Physiol 6: 16–20. 10.1016/j.cophys.2018.03.010 30320243 PMC6181235

[58] Becklake MR, Kauffmann F (1999) Gender differences in airway behaviour over the human life span. Thorax 54: 1119–1138. 10.1136/thx.54.12.1119 10567633 PMC1763756

[59] Fu L, Freishtat RJ, Gordish-Dressman H, (2014) Natural progression of childhood asthma symptoms and strong influence of sex and puberty. Ann Am Thorac Soc 11: 898–907. 10.1513/AnnalsATS.201402-084OC 24896645 PMC4213994

[60] Carey MA, Card JW, Voltz JW, (2007) It’s all about sex: gender, lung development and lung disease. Trends Endocrin Met 18: 308–313. 10.1016/j.tem.2007.08.003 PMC2391086 17764971

[61] DunnGalvin A, Hourihane JOB, Frewer L, (2006) Incorporating a gender dimension in food allergy research: A review. Allergy 61: 1336–1343. 10.1111/j.1398-9995.2006.01181.x 17002711

[62] Agarwal AK, Shah A (1997) Menstrual-linked asthma. J Asthma 34: 539–545. 10.3109/02770909709055398 9428300

[63] Murphy VE, Clifton VL, Gibson PG (2006) Asthma exacerbations during pregnancy: Incidence and association with adverse pregnancy outcomes. Thorax 61: 169–176. 10.1136/thx.2005.049718 16443708 PMC2104591

[64] Rao CK, Moore CG, Bleecker E, (2013) Characteristics of perimenstrual asthma and its relation to asthma severity and control: Data from the Severe Asthma Research Program. Chest 143: 984–992. 10.1378/chest.12-0973 23632943 PMC3747720

[65] Schatz M, Harden K, Forsythe A, (1988) The course of asthma during pregnancy, post partum, and with successive pregnancies: A prospective analysis. J Allergy Clin Immun 81: 495–504. 10.1016/0091-6749(88)90187-X 3346481

[66] Shames RS, Heilbron DC, Janson SL, (1998) Clinical differences among women with and without self-reported perimenstrual asthma. Ann Allerg Asthma Im 81: 65–72. 10.1016/S1081-1206(10)63111-0 9690575

[67] Stenius-Aarniala B, Piirila P, Teramo K (1988) Asthma and pregnancy: a prospective study of 198 pregnancies. Thorax 43: 12–18. 10.1136/thx.43.1.12 2895502 PMC461079

[68] Pali-Schöll I, Jensen-Jarolim E (2019) Gender aspects in food allergy. Curr Opin Allergy Clin Immunol 19: 249–255. 10.1097/ACI.0000000000000529 30893085

[69] Bender BYAE, Matthews R (1981) Adverse foods. Brit J Nutr 46: 403–407. 10.1079/BJN19810048 7317338

[70] Burr ML, Merrett TG (1983) Food intolerance: a community survey. Brit J Nutr 49: 217–219. 10.1079/BJN19830028 6830749

[71] Metcalfe D, Sampson H, Simon RA (2008) Food Allergy: Adverse Reactions to Foods and Food Additives, 4 Ed., Malden: Blackwell Pub. 10.1002/9781444300062

[72] Moneret-Vautrin DA, Morisset M (2005) Adult food allergy. Curr Allergy Asthma Rep 5: 80–85. 10.1007/s11882-005-0060-6 15659269

[73] Schäfer T, Böhler E, Ruhdorfer S, (2001) Epidemiology of food allergy/food intolerance in adults: associations with other manifestations of atopy. Allergy 56: 1172–1179. 10.1034/j.1398-9995.2001.00196.x 11736746

[74] Jansen JJ, Kardinaal AF, Huijbers G, (1994) Prevalence of food allergy and intolerance in the adult Dutch population. J Allergy Clin Immun 93: 446–456. 10.1016/0091-6749(94)90353-0 8120272

[75] Young E, Stoneham MD, Petruckevitch A, (1994) A population study of food intolerance. Lancet 343: 1127–1130. 10.1016/S0140-6736(94)90234-8 7910231

[76] Løvik M, Namork E, Fæste C, (2009) The Norwegian national reporting system and register of severe allergic reactions to food. Nor Epidemiol 14: 155–160. 10.5324/nje.v14i2.238

[77] Kelly C, Gangur V (2009) Sex disparity in food allergy: evidence from the PubMed Database. J Allergy 2009: 1–7. 10.1155/2009/159845 PMC2957586 20975795

[78] Mohammad I, Starskaia I, Nagy T, (2018) Estrogen receptor contributes to T cell-mediated autoimmune inflammation by promoting T cell activation and proliferation. Sci Signal 11: 1–13. 10.1126/scisignal.aap9415 29666308

[79] Heitkemper MM, Chang L (2009) Do fluctuations in ovarian hormones affect gastrointestinal symptoms in women with irritable bowel syndrome? Gend Med 6: 152–167. 10.1016/j.genm.2009.03.004 19406367 PMC3322543

[80] Yung JA, Fuseini H, Newcomb DC (2018) Hormones, sex, and asthma. Ann Allerg Asthma Im 120: 488–494. 10.1016/j.anai.2018.01.016 PMC5936670 29410216

[81] Hall JM, Couse JF, Korach KS (2001) The multifaceted mechanisms of estradiol and estrogen receptor signaling. J Biol Chem 276: 36869–36872. 10.1074/jbc.R100029200 11459850

[82] Ya§ar P, Ayaz G, User SD, (2017) Molecular mechanism of estrogen–estrogen receptor signaling. Reprod Med Biol 16: 4–20. 10.1002/rmb2.12006 29259445 PMC5715874

[83] Kovats S (2015) Estrogen receptors regulate innate immune cells and signaling pathways. Cell Immunol 294: 63–69. 10.1016/j.cellimm.2015.01.018 25682174 PMC4380804

[84] Cunningham M, Gilkeson G (2011) Estrogen receptors in immunity and autoimmunity. Clin Rev Allerg Immu 40: 66–73. 10.1007/s12016-010-8203-5 20352526

[85] Bonds RS, Midoro-Horiuti T (2013) Estrogen effects in allergy and asthma. Curr Opin Allergy Clin Immunol 13: 92–99. 10.1097/ACI.0b013e32835a6dd6 23090385 PMC3537328

[86] Watanabe Y, Tajiki-Nishino R, Tajima H, (2019) Role of estrogen receptors α and β in the development of allergic airway inflammation in mice: A possible involvement of interleukin 33 and eosinophils. Toxicology 411: 93–100. 10.1016/j.tox.2018.11.002 30445053

[87] Graham JH, Yoachim SD, Gould KA (2020) Estrogen receptor alpha signaling is responsible for the female sex bias in the loss of tolerance and immune cell activation induced by the lupus susceptibility locus Sle1b. Front Immunol 11: 582214. 10.3389/fimmu.2020.582214 33240270 PMC7683613

[88] Gandhi VD, Cephus JY, Norlander AE, (2022) Androgen receptor signaling promotes Treg suppressive function during allergic airway inflammation. J Clin Invest 132: e153397. 10.1172/JCI153397 35025767 PMC8843736

[89] Cephus JY, Stier MT, Fuseini H, (2017) Testosterone attenuates group 2 innate lymphoid cell-mediated airway inflammation. Cell Rep 21: 2487–2499. 10.1016/j.celrep.2017.10.110 29186686 PMC5731254

[90] Laffont S, Blanquart E, Savignac M, (2017) Androgen signaling negatively controls group 2 innate lymphoid cells. J Exp Med 214: 1581–1592. 10.1084/jem.20161807 28484078 PMC5461006

[91] Markle JG, Fish EN (2014) SeXX matters in immunity. Trends Immunol 35: 97–104. 10.1016/j.it.2013.10.006 24239225

[92] Kissick HT, Sanda MG, Dunn LK, (2014) Androgens alter T-cell immunity by inhibiting T-helper 1 differentiation. P Natl Acad Sci USA 111: 9887–9892. 10.1073/pnas.1402468111 PMC4103356 24958858

[93] Trigunaite A, Dimo J, Jørgensen TN (2015) Suppressive effects of androgens on the immune system. Cell Immunol 294: 87–94. 10.1016/j.cellimm.2015.02.004 25708485

[94] García-Gómez E, González-Pedrajo B, Camacho-Arroyo I (2013) Role of sex steroid hormones in bacterial–host interactions. Biomed Res Int 2013: 928290. 10.1155/2013/928290 23509808 PMC3591248

[95] Meier A, Chang JJ, Chan ES, (2009) Sex differences in the Toll-like receptor-mediated response of plasmacytoid dendritic cells to HIV-1. Nat Med 15: 955–959. 10.1038/nm.2004 19597505 PMC2821111

[96] Afshan G, Afzal N, Qureshi S (2012) CD4+CD25(hi) regulatory T cells in healthy males and females mediate gender difference in the prevalence of autoimmune diseases. Clin Lab 58: 567–571. 22783590

[97] Fuseini H, Yung JA, Cephus JY, (2018) Testosterone decreases house dust mite-induced type 2 and IL-17A-mediated airway inflammation. J Immunol 201: 1843–1854. 10.4049/jimmunol.1800293 30127088 PMC6143420

[98] Marozkina N, Zein J, DeBoer MD, (2019) Dehydroepiandrosterone supplementation may benefit women with asthma who have low androgen levels: a pilot study. Pulm Ther 5: 213–220. 10.1007/s41030-019-00101-9 32026412 PMC6967310

[99] Zein J, Gaston B, Bazeley P, (2020) HSD3B1 genotype identifies glucocorticoid responsiveness in severe asthma. P Natl Acad Sci USA 117: 2187–2193. 10.1073/pnas.1918819117 PMC6995013 31932420

[100] Wenzel SE, Robinson CB, Leonard JM, (2010) Nebulized dehydroepiandrosterone-3-sulfate improves asthma control in the moderate-to-severe asthma results of a 6-week, randomized, double-blind, placebo-controlled study. Allergy Asthma Proc 31: 461–471. 10.2500/aap.2010.31.3384 21708057

[101] Borba VV, Zandman-Goddard G, Shoenfeld Y (2018) Prolactin and autoimmunity. Front Immunol 9: 73. 10.3389/fimmu.2018.00073 29483903 PMC5816039

[102] Moulton VR (2018) Sex hormones in acquired immunity and autoimmune disease. Front Immunol 9: 2279. 10.3389/fimmu.2018.02279 30337927 PMC6180207

[103] Ngo ST, Steyn FJ, McCombe PA (2014) Gender differences in autoimmune disease. Front Neuroendocrin 35: 347–369. 10.1016/j.yfrne.2014.04.004 24793874

[104] Keselman A, Fang X, White PB, (2017) Estrogen signaling contributes to sex differences in macrophage polarization during asthma. J Immunol 199: 1573–1583. 10.4049/jimmunol.1601975 28760880 PMC5576568

